# Predicting the Success of International Pharmacists in a Distance-Based US Doctor of Pharmacy (PharmD) Program: Results from a 5-Year Cohort

**DOI:** 10.3390/pharmacy10050129

**Published:** 2022-10-08

**Authors:** Paul M. Reynolds, Ralph J. Altiere, Kari L. Franson, Tina P. Brock, Jodie V. Malhotra, Rachel Wagmaister, Shaun Ellen Gleason

**Affiliations:** 1 School of Pharmacy and Pharmaceutical Sciences, University of Colorado Skaggs, 12850 E Montview Blvd, V20 1116G, Aurora, CO 12850, USA; 2 Titus Family Department of Clinical Pharmacy, University of Southern California School of Pharmacy, 1985 Zonal Ave, Los Angeles, CA 90089, USA

**Keywords:** education, pharmacy, academic performance, education, distance, health education, school admission criteria, international educational exchange, global health

## Abstract

Background: To establish the predictors of success in an international-trained PharmD (ITPD) program between admission criteria and academic performance. Methods: The primary outcome of this study was the correlation of admission criteria with didactic and experiential grade point averages (GPA) for the first 5 years. Candidates meeting the minimum criteria completed a competency exam or the US-Foreign Pharmacy Graduate Equivalency Exam (US-FPGEE). Tests of English language proficiency (TOEFL(R) and ACTFL’s Oral Proficiency Interview) plus interview with faculty, students, and alumni were also required. Scores were correlated with both didactic and experiential GPAs. Results: The 23 students admitted to the ITPD program had a cumulative GPA of 3.72. There was a significant correlation between total admissions score and the median pharmacy and healthcare course category GPA (ρ 0.53), but not other categories. The composite TOEFL did not predict any performance but TOEFL writing and speaking did correlate with advanced pharmacy practice experience (APPE) performance. The OPI scores were associated with higher GPAs overall, in advanced integrated clinical sciences, and APPEs. The admission interview scores consistently and significantly correlated with preceptor-rated APPE GPA, practitioner skills, and professionalism (ρ > 0.5; *p* < 0.05). Performance in early courses significantly predicted the performance in advanced courses and experiential performance (ρ 0.48–0.61). Conclusion: The correlations between early and late course performance demonstrated the cohesiveness of this program. Further study is needed between the predictors of success using non-cognitive admission criteria.

## 1. Introduction

As the profession of pharmacy continues to evolve, it becomes imperative that pharmacy education considers approaches that diversify the profession, globalize pharmacists’ patient care, and supply advanced pharmacy practitioners that meet the needs of local healthcare communities across the world [1,2]. Although the pharmacy profession is undergoing robust global growth, the need for well-trained guides for supporting access to the use of quality medicines worldwide is high [3]. These disparities are magnified where advanced training is necessary in order for pharmacists to participate in roles related to direct patient care. In response, multiple pharmacy organizations are offering guidance to the profession regarding career advancement, workforce development, and educational methods [3,4]. Yet, disparities remain between those institutions that have already developed resources for advanced training for the provision of pharmacists’ patient care and those in need of trainers for this type of education [5]. Further study is needed regarding the effective implementation of educational programs that embrace the advancement of pharmacists’ patient care on an international scale, and predictors of trainee success in these programs.

The Accreditation Council for Pharmacy Education (ACPE)-accredited International-Trained PharmD (ITPD) program was established in 2014 at the University of Colorado Skaggs School of Pharmacy and Pharmaceutical Sciences (CU SSPPS) to address the need for pharmacists’ patient care training internationally. This advanced-standing entry-level Doctor of Pharmacy (PharmD) program provides a combination of didactic and experiential course work designed for pharmacists educated and practicing outside of the United States. The overarching goal of this program is to encourage the advancement of pharmacists’ patient care in the student’s home country.

In accordance with the 2016 ACPE accreditation standard 17 appendix 3, PharmD programs should conduct correlations between admission variables and academic performance [6]. In addition, prior studies have suggested that admissions processes should be tailored to predict academic performance in both the didactic and the experiential settings [7]. Given the design of the ITPD program, and the global backgrounds of its students, the purpose of this study is to establish predictors of academic performance and student success in the ITPD program by examining the relationship between admission criteria and performance in both didactic and experiential training. By assuring student success, this program is designed to meet healthcare disparities by generating practice leaders and pharmacy educators who can advocate for the healthcare needs of their respective communities.

## 2. Materials and Methods

### 2.1. Curricular Design and Admission Criteria

The ITPD curriculum of the CU SSPPS was designed as an advanced-standing entry-level PharmD curricular pathway for practicing pharmacists from around the world who aim to advance pharmacists’ patient care in their local healthcare communities. While a separate pathway, it is aligned with the school’s traditional PharmD pathway by sharing ability-based outcomes, faculty members, and a majority of the didactic content. The curriculum includes 90 semester credit hours delivered in a hybrid format. This encompasses online didactic classwork and two in-person sessions of interactive didactic classes that correspond with elements of the experiential curriculum. It also includes in-person introductory pharmacy practice experiences (IPPEs), an advanced introductory pharmacy practice experience (aIPPE), and advanced pharmacy practice experiences (APPEs). As the program is designed to be flexible, time from program admission to completion spans 3 to 6 years. Prior publications have detailed success in tailoring educational methodologies to learning outcomes in this program [8,9].

Advanced standing into the program is assured through requiring a baccalaureate degree (or higher) in pharmacy and that candidates have practiced as a pharmacist for at least one year. Applicants also have to successfully pass two foundational competency exams in biomedical and pharmaceutical sciences. These exams align with coursework commonly covered in baccalaureate pharmacy programs and the first and second years of the CU SSPPS entry-level Doctor of Pharmacy Program. They include topics such as: biochemistry, cell biology, mechanisms of disease, pharmaceutics, medicinal chemistry, microbiology, pharmacology/toxicology, pharmacokinetics, drug design and drug action, immunology, and basic anatomy. In place of these exams, applicants may demonstrate competency via the passing of the US-Foreign Pharmacy Graduate Equivalency Exam (US-FPGEE).

The ITPD applicants are required to demonstrate English language proficiency through the Educational Testing Service’s Test of English as a Foreign Language (TOEFL^®^) exam and the American Council on the Teaching of Foreign Languages (ACTFL) oral proficiency interview (OPI) exam. Upon meeting these qualifications, applicants are invited to video-conference interviews with multiple faculty members, a current ITPD student, and an ITPD alum. Interviews assess methods of learning and researching, critical thinking and decision making, adhering to principles and values of the profession, commitment to pharmacists’ patient care in their home country, and communication and interpretive abilities (assessing the personal and professional domain). These were assessed using a quantitative and qualitative standardized rubric. Scores on each category ranged from 0 to 3 and 0 to 9 through an internally created rubric, with the final interview score calculated as a percentage of possible points, and the multiple interviewer scores averaged. In addition to numeric score, each interviewer provided a qualitative rationale for scoring as well as a qualitative overall impression of each candidate, which were reviewed with candidate scores through our admissions committee.

The admissions score was derived from our admissions process for all our programmatic pathways and was in alignment with ACPE admissions standards regarding the standardized interview process [6]. The admission score was calculated using a sum of the above-mentioned criteria, with TOEFL composite and OPI scores each weighted at 20 points, and the foundational competency exams percentage weighted as 20 points for the biomedical sciences and 30 points for pharmaceutical sciences. If the FPGEE was used in place of the competency exams, the passing percentage was calculated on a 50-point scale. The mean interview score was valued at 100 points. Additional points were offered for preferred criteria of Board of Pharmacy Specialties (BPS) certification, employment at a Joint Commission-accredited institution, being a faculty member at a school/college of pharmacy (all 5 points each), and residency or fellowship training (10 points each).

### 2.2. Outcomes and Statistical Analysis

This study was approved by the Colorado Multi-Institutional Review Board (COMIRB) as a secondary research study with a waiver of informed consent. The primary outcome of this study was the correlation of individualized student quantitative admission criteria with academic performance (both didactic and experiential) completed for the first 5 years of the program’s offering with analysis completed at year 5 of the program’s offering (Summer 2019). The admission criteria evaluated included a cumulative admissions score, TOEFL scores (composite, reading, listening, speaking, and writing), OPI scores, FPGEE scores/performance on biomedical and pharmaceutical sciences competency exams, and interview scores. The academic performance criteria included mean cumulative programmatic grade point average (cGPA) on a 4.0 scale and median grade point average (GPA) within major didactic course categories (detailed in Table 1). These course categories were created on the basis of the required elements of the didactic curriculum for Doctor of Pharmacy degrees as outlined by ACPE [6] with further delineation of major categories into subcategories for the purposes of analysis. Within the experiential curriculum, IPPE and APPE GPA data were included for correlation, along with preceptor assessment of practitioner skills, communication, and professionalism, which determined grades on APPE rotations using previously validated methods [10] and were used to identify potential areas for improvement for skillsets within the didactic curriculum. The secondary outcomes included correlations between academic performance and years of pharmacy practice experience and the comparison of GPA between students employed or not employed at a joint commission accredited facility or presence of a prior graduate degree. Admission criteria were correlated with individual course performance, and the correlation between foundational and advanced course category performance was assessed to determine if performance in foundational courses predicted performance in advanced didactic and experiential courses.

Since a majority of the data were assumed to be non-parametric in nature, a Spearman’s rank-order correlation test was used to assess the strength and significance of each correlation. The strength of correlation was expressed in ρ values, with 0.10–0.39 considered to be a weak correlation, 0.40–0.69 moderate, 0.70–0.89 strong, and >0.90 as very strong [11]. Correlations were considered significant if the *p*-value was less than 0.05 (95% certainty that correlation did not occur due to chance). Correlations that did not express a monotonic relationship were excluded from the analysis. Other comparisons (joint commission employment, prior graduate degree, and academic performance) were conducted using the non-parametric Wilcoxon rank-sum test using the same threshold for statistical significance. All statistics were conducted using JMP Pro Statistical Software (© SAS Institute, Cary, NC, USA).

## 3. Results

### 3.1. Cohort Characteristics and Overall Course Performance

Twenty-three students had been admitted to the ITPD program in its first five years (2014 to 2019). Students earned their original pharmacy degree from 14 unique countries outside of the United States. At the time of the writing of this manuscript, 21 students have graduated and 4 are completing APPE rotations. Two students were unable to continue the program due to non-academic reasons and they were not included in the analysis. At admission, six students had a graduate-level degree and four were employed at Joint Commission-accredited healthcare facilities. The applicants had a mean of 5.5 years (±SD 4.73) of prior pharmacy practice experience.

These students earned a cGPA of 3.72 (±SD 0.31). Median GPA for major course categories was 3.92 (±IQR 0.24) for foundational integrated clinical sciences (fICS); 3.86 (±IQR 0.48) for pharmacy and healthcare; 3.84 (± IQR 0.6) for professionalism and communication and informatics; 3.67 (±IQR 0.36) for advanced integrated clinical sciences (aICS); and 3.52 (+/− IQR 0.67) for integrated clinical sciences-pharmacotherapies (ICS). Experiential GPAs were similar to those of didactic coursework, with a median GPA 4.0 (±IQR 0.2) for IPPEs (including aIPPEs) and 4.0 (± IQR 0) for APPEs.

### 3.2. Correlations between Admissions Criteria and Didactic and Experiential Course Performance

Full results for the assessment of the primary outcome are presented in Table 2 (didactic course performance correlations) and Table 3 (experiential course performance correlations). Within these results, there was a significant correlation between the total admissions score and performance in the pharmacy and healthcare course category (ρ 0.53, *p* = 0.01) but not cumulative cGPA or other course categories (Table 2). Although the TOEFL composite scores were not significantly associated with cGPA or any major course category GPA, TOEFL speaking scores were significantly correlated with APPE GPA (ρ 0.46, *p* = 0.03) and cGPA (ρ 0.48, *p* = 0.03), and TOEFL writing scores were significantly correlated with fICS GPA (ρ 0.47, *p* = 0.03) and APPE practitioner scores (ρ 0.55, *p* = 0.01). Both the TOEFL reading and listening scores, however, were not correlated with any didactic, experiential, or cumulative GPAs. The OPI scores were associated with a higher aICS GPA (ρ 0.48, *p* = 0.02), APPE GPA (ρ 0.55, *p* = 0.01), and a higher cGPA (ρ 0.49, *p* = 0.02). The interview scores were not significantly correlated with any didactic course categories or cGPA but were the strongest predictor for nearly every aspect of APPE, but not IPPE, experiential assessment. The significant correlations included interview scores and APPE GPA (ρ 0.56, *p* = 0.01), APPE practitioner skills (ρ 0.50, *p* = 0.02), and APPE professionalism scores (ρ 0.51, *p* = 0.02) and a non-significant trend toward APPE preceptor rating of communication (ρ 0.39, *p* = 0.09).

### 3.3. Correlations between Foundational Competency Exam, FPGE, and Academic Performance

When considering assessments of advanced standing, the foundational competency exams showed limited correlation with academic performance. The performance on the biomedical sciences competency exam had no correlation with any didactic or experiential course, or cumulative GPA, while the pharmaceutical sciences competency exam scores had a significant negative correlation with APPE preceptor ratings of a student’s practitioner skills (ρ −0.67, *p* = 0.01) and communication (ρ −0.56, *p* = 0.03). Of students who took the FPGEE (*n* = 5), there was a very strong and significant correlation between FPGEE score and foundational and integrated sciences GPA (ρ 0.92, *p* = 0.03) and cGPA (ρ 0.97, *p* = 0.01). The interview scores were not significantly correlated with performance in any major didactic course category GPA or cGPA. In addition, performance on the biomedical sciences and pharmaceutical sciences competency exams did not predict cumulative GPA or course category GPAs.

### 3.4. Predictors of Performance within the Program

Assessment of secondary outcomes demonstrated that years of past experience had no correlation with GPA of any major didactic or experiential course categories with the exception of a negative correlation between past experience and the pharmacy and healthcare GPA (ρ −0.43, *p* = 0.05). The presence of a previous graduate degree (*p* = 0.16) or employment at an international joint commission certified health care facility (*p* = 0.18) was not associated with a significant difference in cGPA. However, students who had prior graduate degrees performed significantly better in the aICS course category compared with those who did not have a prior graduate degree (3.96 median GPA vs. 3.58, *p* = 0.04). A similar trend was noted among the pharmacy and healthcare course category and prior graduate degree (4.0 median GPA vs. 3.67, *p* = 0.01). Conversely, students employed at joint commission certified facilities performed significantly lower in advanced ICS courses vs. those who were not employed at such an institution (3.34 median GPA vs. 3.75, *p* = 0.05). Employment at an international joint commission certified facility or the presence of a prior graduate degree did not significantly predict any experiential grade performance (*p* > 0.05).

### 3.5. Individual Course Analysis

In terms of individual courses, total admissions score also predicted higher performance in the infectious diseases course (ρ 0.56, *p* = 0.01), interprofessional ethics (ρ 0.5, *p* = 0.02), public health (ρ 0.48, *p* = 0.03), instructional methods (ρ 0.58, *p* = 0.01), professional skills development (ρ 0.44, *p* = 0.05), and management and leadership (ρ 0.48, *p* = 0.0267). The TOEFL composite score significantly predicted higher performance in management and leadership (ρ 0.48, *p* = 0.03), communication (ρ 0.5, *p* = 0.02), clinical skills foundations (ρ 0.64, *p* < 0.01), clinical reasoning and decision-making (ρ 0.54, *p* = 0.011), and clinical capstone (ρ 0.43, *p* = 0.046). Within the individual components of the TOEFL exam, TOEFL writing scores predicted success in communication (ρ 0.48, *p* = 0.03), drug information (ρ 0.46, *p* = 0.04), clinical skills foundations (ρ 0.48, *p* = 0.02), capstone (ρ 0.54, *p* = 0.01), and professional skills development (ρ 0.46, *p* = 0.04). TOEFL speaking predicted performance in management and leadership (ρ 0.47, *p* = 0.03), communications (ρ 0.48, *p* = 0.02), GI/Nutrition (ρ 0.46, *p* = 0.04), and clinical reasoning and decision making (ρ 0.56, *p* = 0.01). OPI score significantly predicted higher performance in both the pharmacotherapy of endocrine, hematological, pulmonary, urological disorders and women’s health course (ρ 0.45, *p* = 0.04) and the clinical capstone course (ρ 0.47, *p* = 0.03).

### 3.6. Assessment of ITPD Program Cohesiveness

Once admitted to the program, higher performance in the ITPD foundational course categories significantly predicted success in advanced courses and the experiential curriculum (Table 4). The performance in foundational integrated sciences significantly predicted performance in integrated clinical sciences courses (ρ 0.73, *p* < 0.01) and advanced integrated clinical sciences (ρ 0.64, *p* < 0.01) Similar significant correlations were noted for the pharmacy and healthcare courses and pharmacotherapy courses (ρ 0.63, *p* < 0.01) as well as the advanced pharmacotherapy courses (ρ 0.53, *p* = 0.014). Significant correlations were also noted between the communications and informatics courses and pharmacotherapy courses (ρ 0.8, *p* = ≤ 0.01), and the advanced pharmacotherapy courses (ρ 0.66, *p* < 0.01). IPPE experiential GPA was significantly correlated with performance in the course categories of foundational integrated sciences (ρ 0.46, *p* = 0.04) and advanced integrated clinical sciences (ρ 0.46, *p* = 0.04). The performance in all major didactic course categories correlated well with APPE rotation performance (ρ 0.48–0.61, *p* < 0.02 for all categories).

## 4. Discussion

Establishing effective admission criteria is essential to the success and function of a pharmacy program. Prior pharmacy school admissions studies have shown that traditional cognitive measures (e.g., achievement tests, prior GPA) had strength of correlation of less than 0.30 with didactic performance [7,12,13]. Admissions variables in the cognitive domain are even less predictive for performance in healthcare experiential programs, suggesting the need to bolster measures of non-cognitive criteria such as communication, empathy, citizenship, ethical behavior, commitment to leadership, and diversity [7]. Creation of the multiple mini-interview process has advantages in that it increases the validity of interview scoring in a multicultural context, which overlaps with many aspects of our institution’s interview processes. As the diversity of pharmacy programs continues to grow, a multifaceted and multicultural admissions approach that integrates aspects of non-cognitive criteria in addition to cognitive measures will become imperative for predicting student performance and respective areas for targeted improvement within an admitted cohort of pharmacy students.

Our results suggest that the select aspects of our admissions criteria predict performance in specific aspects of the curriculum as opposed to a holistic prediction of cumulative GPA in an international group of pharmacy students. For example, there was a very strong and significant correlation between performance on standardized testing (FPGEE) score and GPAs in the program. Our non-cognitive criteria, such as interview scores, were one of the strongest factors predicting success in the experiential component of our program. This reflects the need for assessment of these “soft” non-cognitive skills as part of the admissions process and substantiates prior literature suggesting these criteria may better predict experiential performance [7]. Importantly, these held true in this global population with varied pharmacy education and professional backgrounds. Prior studies have also demonstrated pre-pharmacy, science-related GPAs are less likely to correlate with PCAT and NAPLEX performance as opposed to didactic GPA in pharmacy school, supporting our findings of a lack of correlation between our basic science admission exams and pharmacy school GPA once our students entered the program [14]. This study also demonstrated that early pharmacy school GPAs predict late pharmacy school performance (PCOA, NAPLEX) which is similar to our findings that demonstrated that strong early program performance predicted strong late program performance [14]. Other studies have demonstrated a stronger correlation between pre-pharmacy GPA and programmatic success, but students were also more likely to be successful with pre-pharmacy BS degrees and advanced biology course work which is similar to our cohort of ITPD students [12] but these results may not strongly predict NAPLEX performance [15]. The results of this study have informed our program of the importance of maintaining a multi-panel interview during the admissions process, has emphasized the importance of maintaining the OPI for candidates that require this interview, and has provided information to our programs regarding establishing TOEFL cutoffs for our admissions process as well as establishing needs for early interventions in order to improve experiential readiness.

The reasoning for the variability we observed in our correlations may be multifaceted. These results may reflect the complexity of the profession, requiring clinicians to operate on high levels of critical thinking, data integration, and communication to successfully practice. Results from a previous study have suggested testing of English language understanding does not predict academic success within a program in non-native English-speaking students. That study is limited in scope as it solely evaluated composite TOEFL and non-native English-speaking students who came from only one geographic region [16]. Our results are from a more diverse cohort and support the importance of English proficiency as students progress to courses that require greater critical thinking, data integration, and interprofessional communication, such as in our aICS and experiential courses.

Our results also suggest that, overall, our weighted admissions score was unable to predict success across all aspects of our program. Yet, our broad criteria are able to identify global pharmacy professional applicants who can succeed in a US-based PharmD program. This may be reflective of not only our admission criteria, but also the common foundational pharmacy education and motivation of the individuals successfully admitted. The correlations between both early and late didactic courses reflect the educational cohesiveness of our program. Some of the weaker correlations between didactic coursework and experiential performance suggest that skillsets in the classroom may not fully translate to a practice-based setting, but predictive ability did remain valid for major course categories. This is consistent with prior research suggesting that targeted assessments and situational judgment testing are more likely to predict experiential readiness than GPA or pre-pharmacy performance (PCAT or pre-pharmacy GPA) [17].

This study is met with several limitations. We could not perform a multivariable regression due to the smaller sample size of this study, prohibiting the number of predictor variables for an effective regression model and as such, our correlations should be considered associative in nature. Secondly, because ITPD students performed exceedingly well in the program, there was a small degree of variance in cumulative GPA. This is mirrored by prior studies showing exceptional performance of non-traditional PharmD pathways [18]. This, combined with limited power, may have prevented statistical significance of many of the correlations seen in this study. In addition, the multiple comparisons conducted for this study increase the likelihood of experiment-wise error and may have confounded some of the significant findings in this study. However, the emphasis of this study was to examine the predictors of programmatic success in an international cohort of learners, and this study was designed to be as comprehensive as possible to delineate potential targets for programmatic improvement. We acknowledge that these results present signals for further potential research and programmatic change but are associative in nature. Although our admissions standards and scoring rubrics have been used successfully at our institution, indicate programmatic success, and are based on prior publications [12,13,14,15,16,17,18], validation of our rubric and admissions process external to our institution would improve the external validity of our results and may explain lack of correlation between our cumulative score and specific academic performance. As previous studies have outlined the profound impact of a PharmD degree on nontraditional graduate careers, we look forward to following this cohort of students and their career achievements as they contribute to patient-centered care worldwide.

## 5. Conclusions

This study demonstrated that specific aspects of the TOEFL, OPI, and other admission criteria predicted academic success in didactic coursework for the ITPD program. This study adds to the literature regarding predictors of programmatic success in the didactic and experiential setting in a multicultural student population. Further research is needed regarding the predictors of academic success in healthcare programs with international student populations.

## Figures and Tables

**Table 1:** Composure and Credit Allocation of the Didactic International-Trained PharmD Curriculum.

Course (ACPE Element of Didactic Curriculum)	Credit Hours
Professional Communication and Informatics (Social/Administrative/Behavioral Sciences)	
Drug information fundamentals	1
Evidence Based Medicine	3
Instructional Methods	1.5
Healthcare Informatics I and II	2
Pharmacy and Healthcare (Social/Administrative/Behavioral Sciences and Clinical Sciences)	
Pharmacy practice fundamentals	2
Public Health	1
Healthcare economics	1
Interprofessional Educational Development	1
Law	1.5
Leadership and Management	2
Foundational Integrated Clinical Sciences (Clinical Sciences)	
Patient-Centered Communications	2.5
Medical Terminology	0.5
Clinical Skills Foundations	2
Patient-Centered Self-Care	2.5
Foundational Integrated Clinical Sciences—Pharmacotherapies (Clinical Sciences)	
Cardiovascular and Renal	2.5
Gastroenterology and Nutrition	2.5
Infectious diseases	2.0
Oncology	2.0
Bone and Connective Tissue Disorders	0.5
Geriatrics, neurology, psychiatry	3.0
Endocrine, hematology, pulmonology, women’s health	3.0
Advanced Integrated Clinical Sciences (Clinical Sciences)	
Pharmacogenomics	1
Clinical reasoning and decision making	2
Clinical capstone	3.5

**Table 2:** Correlations between Admissions Criteria and Didactic Course Performance.

Course Category GPA or Cumulative GPA	Strength of Correlation between Admission Criterion and GPA for Course Category (ρ)	Statistical Significance (*p*-Value)
Cumulative Total Admissions Score		
Professional Communication and Informatics	0.23	0.31
Pharmacy and Healthcare	0.53	0.01
Foundational Integrated Clinical Sciences	0.08	0.73
Integrated Clinical Sciences	0.16	0.50
Advanced Integrated Clinical Sciences	0.39	0.08
Cumulative GPA	0.19	0.42
TOEFL: Composite		
Professional Communication and Informatics	0.28	0.22
Pharmacy and Healthcare	0.11	0.63
Foundational Integrated Clinical Sciences	0.28	0.20
Integrated Clinical Sciences	0.28	0.22
Advanced Integrated Clinical Sciences	0.34	0.13
Cumulative GPA	0.29	0.2
TOEFL: Speaking		
Professional Communication and Informatics	0.26	0.25
Pharmacy and Healthcare	0.17	0.48
Foundational Integrated Clinical Sciences	0.41	0.06
Integrated Clinical Sciences	0.33	0.14
Advanced Integrated Clinical Sciences	0.34	0.12
Cumulative GPA	0.48	0.03
TOEFL: Writing		
Professional Communication and Informatics	0.38	0.09
Pharmacy and Healthcare	0.11	0.63
Foundational Integrated Clinical Sciences	0.47	0.03
Integrated Clinical Sciences	0.24	0.29
Advanced Integrated Clinical Sciences	0.4	0.07
Cumulative GPA	0.36	0.1
TOEFL: Reading		
Professional Communication and Informatics	−0.2	0.38
Pharmacy and Healthcare	−0.08	0.72
Foundational Integrated Clinical Sciences	−0.16	0.50
Integrated Clinical Sciences	−0.19	0.40
Advanced Integrated Clinical Sciences	−0.03	0.88
Cumulative GPA	−0.17	0.47
TOEFL: Listening		
Professional Communication and Informatics	0.02	0.92
Pharmacy and Healthcare	−0.27	0.24
Foundational Integrated Clinical Sciences	−0.02	0.93
Integrated Clinical Sciences	0.03	0.91
Advanced Integrated Clinical Sciences	0.11	0.63
Cumulative GPA	−0.05	0.84
Oral Proficiency Interview		
Professional Communication and Informatics	0.31	0.17
Pharmacy and Healthcare	0.26	0.24
Foundational Integrated Clinical Sciences	0.36	0.11
Integrated Clinical Sciences	0.35	0.11
Advanced Integrated Clinical Sciences	0.48	0.02
Cumulative GPA	0.49	0.02
Interview Scores		
Professional Communication and Informatics	0.21	0.36
Pharmacy and Healthcare	0.22	0.33
Foundational Integrated Clinical Sciences	0.25	0.27
Integrated Clinical Sciences	0.25	0.74
Advanced Integrated Clinical Sciences	0.17	0.47
Cumulative GPA	0.28	0.21
Foundational Pharmaceutical Sciences Competency Exam		
Professional Communication and Informatics	0.37	0.17
Pharmacy and Healthcare	−0.15	0.60
Foundational Integrated Clinical Sciences	0.35	0.21
Integrated Clinical Sciences	0.32	0.25
Advanced Integrated Clinical Sciences	0.20	0.48
Cumulative GPA	0.39	0.16
Biomedical Sciences Competency Exam		
Professional Communication and Informatics	−0.20	0.45
Pharmacy and Healthcare	−0.33	0.22
Foundational Integrated Clinical Sciences	0.03	0.91
Integrated Clinical Sciences	−0.17	0.53
Advanced Integrated Clinical Sciences	0.12	0.67
Cumulative GPA	−0.02	0.94
FPGEE		
Professional Communication and Informatics	0.82	0.09
Pharmacy and Healthcare	0.82	0.09
Foundational Integrated Clinical Sciences	0.92	0.03
Integrated Clinical Sciences	0.87	0.05
Advanced Integrated Clinical Sciences	0.56	0.32
Cumulative GPA	0.97	<0.01

**Table 3:** Correlations between Admissions Criteria and Experiential Performance.

Experiential Course Category GPA or Preceptor Performance Rating	Strength of Correlation between Admission Criterion and GPA or Preceptor Rating for Course Category (ρ)	Statistical Significance (*p*-Value)
Cumulative Total Admissions Score		
IPPE GPA	−0.10	0.66
APPE GPA	0.21	0.34
APPE—Practitioner Skills	0.31	0.18
APPE—Professionalism	0.43	0.06
APPE—Communication	0.3	0.22
TOEFL: Composite		
IPPE GPA	−0.01	0.98
APPE GPA	0.25	0.27
APPE—Practitioner Skills	−0.01	0.95
APPE—Professionalism	−0.13	0.59
APPE—Communication	0.05	0.83
TOEFL: Speaking		
IPPE GPA	0.26	0.33
APPE GPA	0.46	0.03
APPE—Practitioner Skills	0.21	0.38
APPE—Professionalism	0.17	0.48
APPE—Communication	0.19	0.43
TOEFL: Writing		
IPPE GPA	0.26	0.33
APPE GPA	0.13	0.58
APPE—Practitioner Skills	0.55	0.01
APPE—Professionalism	0.19	0.42
APPE—Communication	0.17	0.46
TOEFL: Reading		
IPPE GPA	−0.01	0.98
APPE GPA	0.14	0.53
APPE—Practitioner Skills	0.14	0.57
APPE—Professionalism	−0.07	0.75
APPE—Communication	0.18	0.44
TOEFL: Listening		
IPPE GPA	−0.10	0.72
APPE GPA	−0.24	0.3
APPE—Practitioner Skills	−0.40	0.08
APPE—Professionalism	−0.40	0.07
APPE—Communication	−0.26	0.27
Oral Proficiency Interview		
IPPE GPA	0.41	0.06
APPE GPA	0.55	0.01
APPE—Practitioner Skills	0.08	0.72
APPE—Professionalism	0.06	0.8
APPE—Communication	0.19	0.41
Interview Scores		
IPPE GPA	0.35	0.11
APPE GPA	0.56	0.01
APPE—Practitioner Skills	0.5	0.02
APPE—Professionalism	0.51	0.02
APPE—Communication	0.39	0.09
Foundational Sciences Competency Exam		
IPPE GPA	−0.22	0.41
APPE GPA	−0.18	0.5
APPE—Practitioner Skills	−0.67	0.01
APPE—Professionalism	−0.28	0.29
APPE—Communication	−0.55	0.03
Biomedical Sciences Competency Exam		
IPPE GPA	−0.14	0.62
APPE GPA	0.11	0.67
APPE—Practitioner Skills	−0.18	0.51
APPE—Professionalism	−0.24	0.37
APPE—Communication	−0.08	0.74
FPGEE Score		
IPPE GPA	N/A ^1^	N/A ^1^
APPE GPA	0.50	0.67
APPE—Practitioner Skills	0.50	0.67
APPE—Professionalism	0.86	0.30
APPE—Communication	0.50	0.67

^1^ sample size too small to generate correlation.

**Table 4:** Correlations between Foundational Course Performance and Advanced Course Performance.

Earlier Course	Late Course	Strength of Correlation (ρ)	*p*-Value
Correlations Between Early and Late Didactic Course Work			
Foundational Integrated Sciences	Integrated Clinical Sciences	0.79	<0.01
Foundational Integrated Sciences	Advanced Integrated Clinical Sciences	0.64	0.01
Foundational Integrated Sciences	Communication and Informatics	0.82	<0.01
Foundational Integrated Sciences	Pharmacy and Healthcare	0.58	<0.01
Communication and Informatics	Integrated Clinical Sciences	0.67	<0.01
Communication and Informatics	Advanced Integrated Clinical Sciences	0.66	<0.01
Integrated Clinical Sciences	Advanced Integrated Clinical Sciences	0.73	<0.01
Correlations between Didactic and Experiential Course Work			
Foundational Integrated Sciences	IPPE Course GPA	0.45	0.04
Foundational Integrated Sciences	APPE Course GPA	0.64	<0.01
Foundational Integrated Sciences	APPE Communication Score	0.24	0.29
Foundational Integrated Sciences	APPE Professionalism Score	0.27	0.24
Foundational Integrated Sciences	APPE Practitioner Skills Score	0.1	0.36
Integrated Clinical Sciences	IPPE Course GPA	0.45	0.03
Integrated Clinical Sciences	APPE Course GPA	0.48	0.02
Integrated Clinical Sciences	APPE Communication Score	0.05	0.82
Integrated Clinical Sciences	APPE Professionalism Score	0.0008	0.99
Integrated Clinical Sciences	APPE Practitioner Skills Score	−0.09	0.69
Communication and Informatics	IPPE Course GPA	0.3	0.18
Communication and Informatics	APPE Course GPA	0.46	0.04
Communication and Informatics	APPE Communication Score	−0.06	0.78
Communication and Informatics	APPE Professionalism Score	0.41	0.07
Communication and Informatics	APPE Practitioner Skills Score	−0.05	0.81
Advanced Integrated Clinical Sciences ^1^	IPPE Course GPA	0.46	0.04
Advanced Integrated Clinical Sciences	APPE Course GPA	0.57	<0.01
Advanced Integrated Clinical Sciences	APPE Communication Score	0.23	0.32
Advanced Integrated Clinical Sciences	APPE Professionalism Score	0.36	0.11
Advanced Integrated Clinical Sciences	APPE Practitioner Skills Score	0.11	0.63

^1^ For this category a majority, but not all, of the coursework preceded IPPE activities.

## Data Availability

The data presented in this study are available on request from the corresponding author. The data are not publicly available due to protection of student privacy.
